# Nitrogen dioxide oxidizes mitochondrial cytochrome *c*

**DOI:** 10.1016/j.freeradbiomed.2011.09.024

**Published:** 2012-01-01

**Authors:** Rebecca S. Silkstone, Maria G. Mason, Peter Nicholls, Chris E. Cooper

**Affiliations:** Department of Biological Sciences, University of Essex, Colchester, Essex, CO4 3SQ UK

**Keywords:** nitric oxide, cytochrome c, nitrogen dioxide, free radical, urate, mitochondria

## Abstract

We previously reported that high micromolar concentrations of nitric oxide were able to oxidize mitochondrial cytochrome *c* at physiological pH, producing nitroxyl anion (Sharpe and Cooper, 1998 Biochem. J. 332, 9–19). However, the subsequent re-evaluation of the redox potential of the NO/NO^-^ couple suggests that this reaction is thermodynamically unfavored. We now show that the oxidation is oxygen-concentration dependent and non stoichiometric. We conclude that the effect is due to an oxidant species produced during the aerobic decay of nitric oxide to nitrite and nitrate. The species is most probably nitrogen dioxide, NO_2_^•^ a well-known biologically active oxidant. A simple kinetic model of NO autoxidation is able to explain the extent of cytochrome *c* oxidation assuming a rate constant of 3 × 10^6^ M^-1^ s^-1^ for the reaction of NO_2_^•^ with ferrocytochrome *c*. The importance of NO_2_^•^ was confirmed by the addition of scavengers such as urate and ferrocyanide. These convert NO_2_^•^ into products (urate radical and ferricyanide) that rapidly oxidize cytochrome *c* and hence greatly enhance the extent of oxidation observed. The present study does not support the previous hypothesis that NO and cytochrome *c* can generate appreciable amounts of nitroxyl ions (NO^-^ or HNO) or of peroxynitrite.

## Graphical abstract

**Figure:**
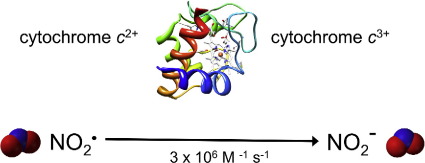

**Highlights:**

► Nitric oxide has been previously reported to oxidize mitochondrial cytochrome *c*. ► We now show that this reaction is oxygen concentration dependent. ► Nitrogen dioxide scavengers (urate; ferrocyanide) modulate the oxidation. ► We construct a kinetic model consistent with nitrogen dioxide as the oxidizing species. ► The model predicts a rate constant for cytochrome *c* oxidation of 3 × 10^6^ M^-1^ s^-1^.

## Introduction

The intercellular messenger nitric oxide has several target molecules in a typical cell. Most but perhaps not all are heme proteins. The main target, guanylate cyclase, is a heme protein whose enzymatic functioning is controlled by NO binding to a heme group in its ferrous state [1]. Another possible target, cytochrome *c* oxidase, can bind NO in both reduced and oxidized states [2], affecting cell respiration in both obvious [3] and more subtle [4] ways. Catalase is unusual in binding NO primarily in its ferric state [5] and suffering consequent inhibition [6]. Cytochrome *c* is another multifunctional cellular heme protein. It is a key component of the mitochondrial respiratory chain, cycling reversibly between ferrous and ferric states; in its ferric [7] but not ferrous [8] state it is also a trigger for programmed cell death (apoptosis). In the ferric state cytochrome *c* also binds NO; the corresponding ferrous–NO complex can be formed only by inducing major structural changes in the molecule, classically by high pH [8] and more recently by binding to cardiolipin [9,10]. Some of these processes, such as those binding to ferrous heme in guanylate cyclase and cytochrome *c* oxidase, and ferric heme in catalase and cytochrome *c*, are reversible. The bound NO can dissociate back to give the original unmodified heme and free NO in solution. Others, including the NO interactions with oxidized cytochrome *c* oxidase, are irreversible. The bound NO is oxidized to nitrite and one of the associated redox centers in the NO binding site is reduced [11].

In 1998 Sharpe and Cooper [12] reexamined the reactions of NO with cytochrome *c*. They were able to confirm the reversible binding of NO to the native protein in the ferric state, with a dissociation constant of about 20 μM. But they also observed a previously undocumented effect of NO on the ferrous cytochrome at neutral pH and in its native state, contrasting with classical observations that NO binding to ferrocytochrome *c* occurs only at extreme pH. They were also able to show a ferrocytochrome *c*-catalyzed increase in the decay rate (autoxidation) of aqueous NO. They interpreted these results in terms of nitroxyl (NO^−^/HNO) production from NO followed by nitroxyl decay.

More recently, however, the redox potential of the NO^•^/NO^−^ couple, previously calculated as more positive than the cytochrome *c* potential [13], has been reanalyzed by two research groups [14,15]. Both agree that the *E*_0_′ for this couple is far more negative than originally estimated. The most accessible couple is NO, H^+^/^1^HNO. Shafirovich and Lymar [14] estimate a reduction potential of − 0.55 V at pH 7. The reduction to the triplet state, ^3^HNO, necessary to form peroxynitrite by reaction with oxygen, is even less favorable.

If these new estimates are correct the NO^•^/NO^−^ couple is highly unlikely to be involved in the NO-induced oxidation of cytochrome *c*. Furthermore in the absence of oxygen, HNO should form N_2_O either via reacting with NO or via dehydrative dimerization. However, attempts to detect N_2_O by adding NO anaerobically to ferrocytochrome *c* were unsuccessful (N. Hogg, Medical College of Wisconsin, Milwaukee, WI, USA, personal communication).

The results of Sharpe and Cooper thus call for a mechanistic reinterpretation. One possible complication is that the observed oxidation could reflect the catalytic activity of a small population of modified cytochrome *c* molecules, possibly polymeric forms [16], which then oxidize the major cytochrome *c* fraction. The second complication is that experiments were done aerobically. This was deliberate, as the action of NO on cytochrome *c* was being compared with the effects of cytochrome *c* on aerobic NO decay and the resultant consequences for the inhibitory effects of NO on cytochrome *c* oxidase activity. However, this creates the possibility that the oxidant is not NO itself but a reactive species derived from NO autoxidation.

Although there is evidence for NO^−^ (HNO) formation in the presence of some heme proteins, the source is typically a strong reductant such as hydroxyurea or cyanamide [17]. Ferricytochrome *c* reacts with HNO to give NO and ferrous cytochrome *c*[18], suggesting the driving force in this system acts in the direction of reduction of cytochrome *c*, not its oxidation. We will show that the most likely cytochrome *c* oxidant is nitrogen dioxide (NO_2_^•^), formed in the autoxidation of NO in the aerobic system used in the assay.

## Materials and methods

Horse heart cytochrome *c* (Sigma; type VI, prepared without the use of trichloroacetic acid) was repurified by cation column chromatography, and the resulting fractions were tested for CO binding and for ascorbate and dithionite reducibility to determine the amounts of any modified fractions of the protein. Cytochrome *c* (100 mg) was dissolved in 1 ml of 100 mM potassium phosphate buffer, pH 7.4, containing 0.1 mM diethylenetriamine pentaacetic acid (DTPA). The cytochrome *c* was passed down a CM52 cation-exchange column equilibrated with 85 mM potassium phosphate, pH 7.4, buffer. Fractions were collected automatically (60 fractions, 10 ml each) and analyzed by optical spectroscopy for the extent of reduction of the ferric form by ascorbate and dithionite. The major repurified cytochrome *c* fraction showed negligible spectrophotometric differences between ascorbate and dithionite reducibility and negligible CO binding to the reduced state. The estimated polymer concentration (from the difference between the *A*_550nm_ and the *A*_540nm_ values for dithionite and ascorbate reductions) was less than 1% of the total. Unless otherwise stated the repurified polymer-free samples were used in all experiments.

Ferrocytochrome *c* was made by the addition of excess ascorbate (50 mM) and the solutions were incubated for 20 min at room temperature. To remove ascorbate the samples were then passed down a Sephadex G-25 column equilibrated with 100 mM potassium phosphate buffer, 0.1 mM DTPA, pH 7.0. The cytochrome *c* concentrations were calculated using a reduced (Fe^2+^) minus oxidized (Fe^3+^) extinction coefficient (*A*_550nm_ − *A*_540nm_) of 21.2 mM^− 1^ cm^− 1^.

Sodium proliNONOate (proliNO) from Aventis Chemicals was used as a rapid-release nitric oxide donor (pure NO gas had similar effects). The concentration of proliNO in solution was determined in 20 mM NaOH using an extinction coefficient at 248 nm (*A*_248nm_) of 8.6 mM^− 1^ cm^− 1^. The ratio of [proliNO] to [NO] released was obtained by titration with oxyhemoglobin (*A*_557nm_ − *A*_630nm_) using a difference extinction coefficient of 14.4 mM^− 1^ cm^− 1^. The stock proliNO sample gave a [proliNO]:[NO]ratio of 1:1.94.

Experiments carried out under anaerobic conditions were achieved using either extensive vacuum degassing or the addition of a glucose/glucose oxidase/catalase mixture. Decay products of proliNO (control) were produced by allowing proliNO to decay in 100 mM potassium phosphate buffer, 0.1 mM DTPA, for 45 min.

Cytochrome *c* reduction was followed using the change in absorbance at 550–540 nm. To convert this to percentage reduction, fully oxidized and fully reduced cytochrome *c* absorbance values were measured before the termination of each experiment by addition of ferricyanide (10 μM K_3_Fe(CN)_6_) and dithionite (10 μl of a 10% solution in 3 ml), respectively. All spectrophotometric data were collected via a Cary 5E optical spectrophotometer (Varian) and analyzed using Microsoft Excel with data-fitting in Kaleidagraph®.

DTPA was used as a metal chelator in all experiments to prevent unwanted redox side reactions from contaminating metals such as iron. DTPA was used at 0.1 mM for the studies illustrated, but varying the concentration from 0.01 to 0.1 mM had no effect on the extent of cytochrome *c* oxidation observed.

Concentrations of dissolved oxygen and nitric oxide were measured in a glass chamber connected to an oxygen electrode with a Digital Model 10 controller (Rank Brothers, Cambridge, UK). The chamber was fitted with a specially adapted flowthrough plunger to accommodate an ISO-NO NO electrode connected to an ISO-NO MK2 NO meter (World Precision Instruments, Stevenage, UK). The NO electrode was calibrated with proliNO under anaerobic conditions (achieved by a glucose/glucose oxidase/catalase mixture). The oxygen and NO electrode systems were connected to a MacLab 8E data acquisition system (AD Instruments) and the stored data analyzed with Microsoft Excel. Optical spectra were collected simultaneously with electrode data using an Ocean Optics CCD-based fiber-optic spectrophotometer in a custom-built spectroelectrode system [19].

Kinetic modeling of differential equations used the Rosenbrock stiff algorithm in the Macintosh computer software Berkeley Madonna (version 8.3.18; copyright Robert I. Macey and George F. Oster); the minimum step size was set to 1 × 10^− 6^ s and the maximum to 1 s, with a tolerance of 0.01.

Unless otherwise stated the term “significant” in the text refers to unpaired *t* tests, with the significance level set at *p* < 0.05.

## Results

**Fig. 1:**
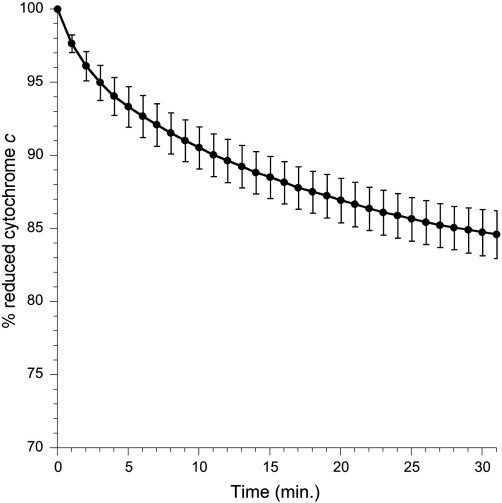
Cytochrome *c* oxidation by proliNO under aerobic conditions. 40 μM proliNO was added to 10 μM ferrocytochrome *c* at a temperature of 30 °C and a pH of 7.4, buffered with 100 mM potassium phosphate, 100 μM DTPA. Cytochrome *c* reduction was calculated as described under Materials and methods. Means ± SD (*n* = 4).

As is the case with NO gas [12], the addition of the fast-releasing NO donor proliNO oxidizes horse heart ferrocytochrome to produce ferricytochrome *c* (Fig. 1). Repurification of the commercial cytochrome sample to remove the small fraction of NO-binding polymers (cf. Materials and methods) had no significant effect on the amount of ferrocytochrome *c* remaining after 30 min (84.0 ± 2.4% for unpurified and 84.7 ± 1.6% for purified; mean ± SD, *n* = 4). Catalysis by polymers is thus not responsible for cytochrome *c* oxidation by NO solutions.

**Fig. 2:**
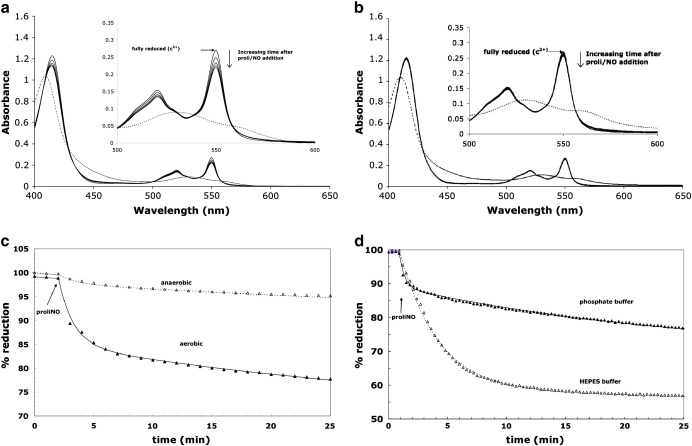
Spectroscopic changes in cytochrome *c* treated with proliNO under aerobic and anaerobic conditions. Conditions were as for Fig. 1. (a) Spectra are shown for 0, 5, 15, and 30 min after the aerobic addition of 40 μM proliNO followed by the addition of 10 μM K_3_Fe(CN)_6_. Dashed lines indicate the spectrum post ferricyanide addition. Subsequent addition of sodium dithionite (0.03% final concentration) produced essentially the same spectrum as that of the initial ferrocytochrome *c*. (b) Same as (a) but under anaerobic conditions. (c) Time courses from (a) and (b); (d) same as (c) but replacing 100 mM potassium phosphate with 100 mM sodium Hepes.

**Fig. 3:**
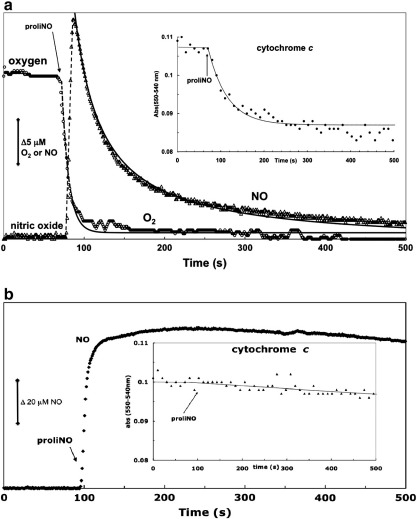
Parallel measurements of oxygen and NO concentrations and the concomitant changes in the redox state of cytochrome *c*. The change in redox state of 10 μM ferrocytochrome *c* was monitored after the addition of a single 40 μM aliquot of proliNO. The medium was a 100 mM potassium phosphate, 100 μM DTPA, pH 7.4, buffer, at 30 °C. (a) Time courses of NO and O_2_ concentrations and (inset) cytochrome *c* redox state under aerobic conditions. (b) Same as (a) but under anaerobic conditions.

It was therefore important to study the effect of oxygen and medium composition. Fig. 2 summarizes the spectral changes occurring under aerobic and anaerobic conditions. Under aerobic conditions (Fig. 2a), oxidation is clearly observed in both the Soret and the visible (550 nm) regions. There is no detectable cytochrome *c* modification other than the oxidation. No oxidation was observed when the decay products of the NO donor were added in place of the proliNO (results not shown). Significantly there was no oxidation of ferrocytochrome *c* under anaerobic conditions (Fig. 2b). NO alone is therefore not responsible for the observed effects. Fig. 2c plots the corresponding absorbance time courses at the wavelength pair 550 nm–540 nm under the two conditions. Aerobic addition of approximately 40 µM proliNO (giving 78 µM NO) induces the oxidation of about 2 μM cytochrome *c* in phosphate buffer, with a time course that tracks the decay of the NO (cf. Figs. 3a and b). There is no significant oxidation under anaerobic conditions.

The extent of the aerobic oxidation is also buffer dependent. In the previous work of Sharpe and Cooper [12], a greater proportional aerobic oxidation of cytochrome *c* was reported compared to our results (see their Figs. 1, 2, and 4). Their buffer system was, however, Hepes-based, whereas we have used phosphate as our default buffer. Fig. 2d compares the NO:cytochrome *c* stoichiometry in the two systems. Increased oxidation is seen with Hepes buffer compared to phosphate.

Using the oxygen and nitric oxide spectroelectrode system it was possible to measure changes in O_2_ and NO levels concurrent with the redox changes in cytochrome *c*. Under aerobic conditions (Fig. 3a), after addition of an excess of proliNO to a solution of ferrocytochrome *c*, there is an immediate NO electrode response showing the appearance of free NO and—at nearly the same time—a decrease in oxygen concentration. The NO level in solution decreases to zero over approximately 2 min. This is accompanied by the oxidation of ferrocytochrome *c* (Fig. 3a, inset). However, under anaerobic conditions the NO level remains high (Fig. 3b) and there is no appreciable decrease over a similar time period. Concomitantly there is no appreciable oxidation of ferrocytochrome *c* (Fig. 3b, inset).

**Fig. 4:**
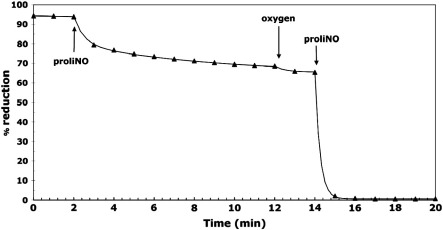
Effects of excess oxygen upon ferrocytochrome *c* oxidation induced by nitric oxide. Each experiment involved two additions of 40 μM proliNO to 10 μM ferrocytochrome *c*. The oxygen concentration in the solution was increased by flushing the cuvette with O_2_ gas before a second addition of proliNO as indicated. Other conditions were as for Fig. 1 (phosphate buffer).

The importance of oxygen was further studied by carrying out experiments in the presence of higher than ambient levels of O_2_ (up to approximately 1 atm or 1 mM O_2_). Fig. 4 illustrates the results obtained at such high oxygen levels. The initial addition of proliNO under ambient O_2_ levels (approximately 230 μM) yields the 20–25% oxidation seen previously. But a subsequent increase in oxygen concentration in the reaction vessel, followed by a second, equal, addition of proliNO gave rise to essentially complete oxidation of cytochrome *c*. The ratio *c*_oxidized_:NO is thus O_2_ dependent. Furthermore, when conditions are initially anaerobic, addition of proliNO does not result in any immediate oxidation, but oxygen injection then leads to a substantial oxidation event (not shown).

These observations resulted in the search for a possible reaction product of NO and oxygen that could be responsible for the observed effect. The initial disappearance of NO in the presence of oxygen has been extensively studied [20–24]. Although the fine details of the mechanism are disputed, all authors agree that NO_2_^•^ is an intermediate. NO_2_^•^ is known to be an active oxidant in biological systems [24,25]. It rapidly oxidizes a number of biological species, including sulfhydryl compounds and uric acid. Conversely, these substances can therefore act as NO_2_^•^ traps, removing it from the system reductively to give nitrite [25–27]. Experiments were therefore conducted using various scavengers, including glutathione and cysteine. However, although there was an apparent prevention of the oxidation of cytochrome *c* by NO, further experiments with these agents in the absence of NO showed that this was actually due to their re-reducing oxidized cytochrome *c* during the reaction (data not shown).

**Fig. 5:**
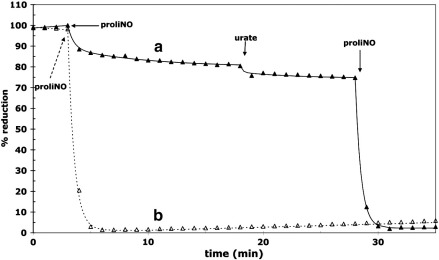
Effect of urate upon the NO-induced oxidation of ferrocytochrome *c*. Each experiment involved addition of 40 μM proliNO to aerobic 10 μM ferrocytochrome *c*. Other conditions were as for Fig. 1 (phosphate buffer). (Trace a) 1.0 mM urate and a second addition of proliNO added at the indicated times. (Trace b) 1.0 mM urate present from start of experiment.

**Fig. 6:**
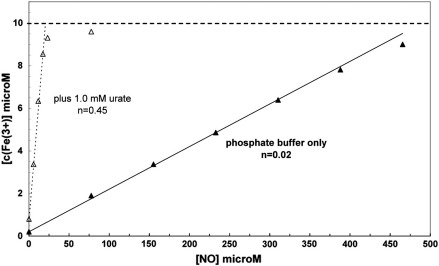
Aerobic titration of ferrocytochrome *c* with proliNO in the presence and absence of urate. The fractional oxidation of the cytochrome is plotted after successive additions of proliNO to 10 μM ferrocytochrome *c* under standard aerobic conditions (as in Fig. 1) and in the presence or absence of 1.0 mM urate. The plot shows micromolar cytochrome *c* oxidized against microequivalents of total NO added. Stoichiometries were calculated from the linear slopes.

Urate, however, is an NO_2_^•^ scavenger that is a less effective cytochrome *c* reductant. Fig. 5 shows the results obtained with the cytochrome *c*–NO system in its presence. Surprisingly, cytochrome *c* is fully oxidized upon addition of NO, either when urate is present in the starting buffer (Fig. 5, trace b) or when urate is added during the course of the reaction (Fig. 5, trace a). Ferrocytochrome *c* was therefore titrated aerobically in the presence and absence of 1.0 mM urate. The results are shown in Fig. 6. This summarizes the progressive oxidation of ferrocytochrome *c* induced by sequential additions of proliNO, plotting the cytochrome *c* oxidation as a function of total NO added. There is no significant change in the magnitude of oxidation with multiple additions of NO although at high levels of cytochrome *c* oxidation some NO reacts with the ferric species to form an NO–ferri complex, as observed by Sharpe and Cooper [12]. Aerobic NO titration in phosphate buffer gives a ratio of cytochrome *c* oxidized to NO added of approximately 0.02. But whereas, in the absence of urate, 40–50 mM NO is required to reduce 1 mM cytochrome *c*, in the presence of urate only about 2.5 mM NO is required. The ratio of cytochrome *c* oxidized to NO added is now between 0.4 and 0.5. The stoichiometry of cytochrome *c* oxidation was not enhanced by increasing urate concentration further; indeed the final extent of cytochrome *c* oxidation began to decrease above 1 mM urate, as re-reduction of ferricytochrome *c* by urate became noticeable (results not shown).

Ferrocyanide is an NO_2_^•^ scavenger [28] whose rate of reduction of cytochrome *c* is unfavored thermodynamically. Attempts to scavenge NO_2_^•^ with ferrocyanide, however, suffer from the same problem as urate in that the product of NO_2_^•^ oxidation—ferricyanide—is readily able to oxidize cytochrome *c*. Therefore, as with urate, the addition of ferrocyanide resulted in enhanced oxidation of ferrocytochrome *c* after NO addition (results not shown).

## Discussion

We have confirmed the experimental findings of Sharpe and Cooper [12] that ferrocytochrome *c* is oxidized in the presence of NO. However, we have shown that the induced cytochrome *c* oxidation has a strong oxygen concentration dependence. The NO effect upon cytochrome *c* cannot therefore be due to NO itself, as originally suggested, but to a secondary species generated during its autoxidation. Possible candidate oxidants produced during NO autoxidation include NO_3_^•^ and N_2_O_3_[20–24]. However, both species can be formed only at very low concentrations compared to NO_2_^•^. N_2_O_3_[22] is also an unlikely candidate because, though effective as a hydroxylating agent [26,27], it does not appear to readily undergo one-electron reduction.

We therefore postulated that the relevant oxidizing species is NO_2_^•^. The effects of NO_2_^•^ scavengers such as urate and ferrocyanide support this conclusion. There is a large redox potential difference between NO_2_^•^ and its ultimate oxidation product nitrite. This is more than enough to thermodynamically drive the oxidation of cytochrome *c*. We have been unable to find an NO_2_^•^ scavenger that neither reduces ferricytochrome *c* nor produces a secondary oxidant that oxidizes ferrocytochrome *c*. So this conclusion is still somewhat provisional, at least until direct pulse radiolysis measurements are made of the rate of NO_2_^•^ reaction with cytochrome *c*.

**Fig. 7:**
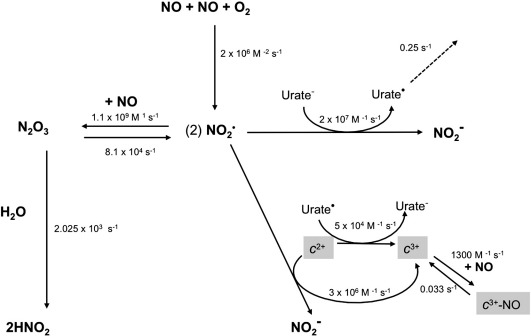
Pathways of nitric oxide autoxidation in an aqueous medium. *c*^2+^/*c*^3+^, ferric/ferrous cytochrome *c*; radical species indicated by superscript dots (^•^). Rate constants were taken from the literature as follows: NO autoxidation pathway values are from Lancaster [13] and references therein; urate oxidation by NO_2_^•^ from Simic and Jovanovin [29] and Ford et al. [34]; the rates for NO binding to oxidized cytochrome *c* were from Sharpe and Cooper [12]; the rate of NO_2_^•^ oxidation of cytochrome *c* was calculated to best fit the data in this paper (see Figs. 8a and 8c); the rate of urate radical oxidation of cytochrome *c* and its uncatalyzed decay to an unspecified product were chosen to be consistent with the data relating to the enhanced oxidation of cytochrome *c* in its presence (see Figs. 8b and 8c)—note that the data allow considerable leeway in the absolute choice of these rate constants though the ratio of the two is more constrained.

**Fig. 8:**
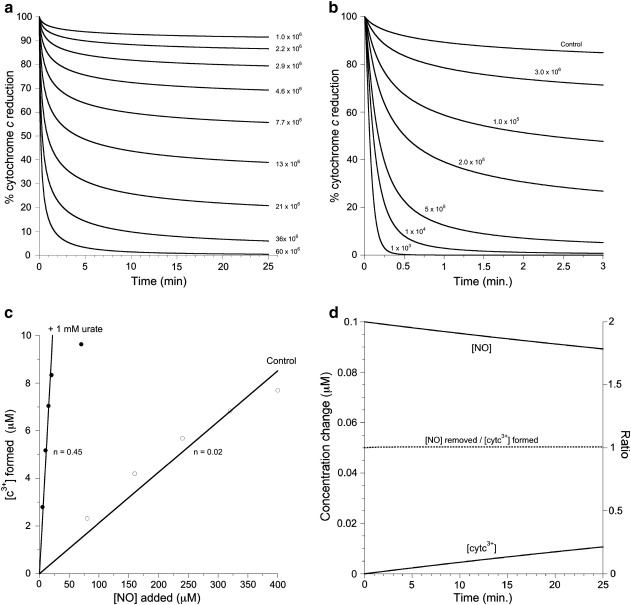
Modeling of cytochrome *c* oxidation by nitrogen dioxide. Simulations were carried out using the rate constants in Fig. 7 unless otherwise indicated. (a) The effect of the addition of 78 µM NO to 10 mM cytochrome *c*, varying the rate constant (M^− 1^ s^− 1^) for the NO_2_^•^ oxidation of cytochrome *c* as indicated. (b) Same as (a) with varying urate concentrations as indicated. (c) The computed concentration of ferricytochrome *c* formed after simulations of repeated boluses of NO in the absence and presence of 1 mM urate. The simulation was allowed to run for 50 min before the next addition of NO. Lines indicate linear regression (with slopes indicated) to all the points in the control data and the linear part of the urate curve, i.e., when the total [NO] < 25 µM. (d) The effects of the addition of 0.5 mM NO to 800 mM ferrocytochrome *c* on the concentrations of NO and ferricytochrome *c* (left axis). The ratio of [NO] removed to ferricytochrome *c* formed is indicated on the right axis. A starting oxygen concentration of 200 µM was used for all simulations. Unless otherwise indicated all other species were set to zero concentration at the start of the simulation.

It is possible, however, to measure the range of possible reaction rates—and whether they are plausible—by kinetic modeling. Fig. 7 indicates a simplified kinetic model for the reactions occurring during NO autoxidation in the presence of cytochrome *c* and urate. The initial limiting step is characterized by the third-order reaction of two molecules of NO with one molecule of oxygen forming two molecules of NO_2_^•^. In the absence of cytochrome *c* this reacts reversibly with another molecule of NO to form N_2_O_3_, which then irreversibly converts to the end product nitrite. We propose that ferrocytochrome *c* can bypass these reactions by reacting directly and irreversibly with NO_2_^•^ to form ferricytochrome *c* and nitrite. Fig. 8a shows the effect of varying the rate of this reaction on the oxidation of ferrocytochrome *c* by an aerobic NO solution. A second-order rate constant on the order of 3 × 10^6^ M^− 1^ s^− 1^ is able to mimic the experimental results seen in this paper.

The effects of urate, ferrocyanide, and Hepes are also readily explainable by this model. The literature values for the relative redox potentials for the ferric/ferrous cytochrome *c* (+ 260 mV) and the urate/urate radical (+ 590 mV) [29] result in a strong driving force for the urate radical oxidation of cytochrome *c*. Incorporating this reaction into the model results in an increased rate and extent of cytochrome *c* oxidation as the urate concentration is varied; Fig. 8b illustrates modeled time courses similar to the experimental data of Fig. 4. The stoichiometry of NO oxidation of cytochrome *c* is also consistent with the data in this paper in the presence and absence of urate (compare Figs. 4 and 8c). At saturating urate concentrations (1 mM) the model predicts essentially 100% trapping of NO_2_^•^. The substoichiometric oxidation of cytochrome *c* in this instance (0.4–0.5) therefore requires either reversibility of the urate radical oxidation of ferrocytochrome *c* or a competing pathway for urate radical decay. We favor the latter explanation, as the rate of the reverse reaction—the urate reduction of ferricytochrome *c*—that was required to model the data resulted in significant reduction of oxidized cytochrome *c* even at low (< 1 mM) urate concentrations. This is not consistent with our experimental data, as the ferricytochrome *c* product was stable under these conditions.

As piperazine compounds such as Hepes can undergo one-electron oxidation to radical intermediates [30], their effects on the extent of cytochrome *c* oxidation are best understood if they act as NO_2_^•^ scavengers, though with considerably less efficiency than ferrocyanide or urate. Assuming all the radical is captured by cytochrome *c*, a rate of NO_2_^•^ scavenging as low as 200 M^− 1^ s^− 1^ would be able to reproduce the results in Fig. 2. However, the exact rate cannot readily be estimated by the model as it is a combination of two unknowns, the rate of Hepes oxidation by NO_2_^•^ and the rate of Hepes radical trapping by ferrocytochrome *c*. As well as Hepes, NO_2_^•^ could oxidize other compounds in the solution to form reducing radicals; these could in turn reduce a small amount of oxygen to superoxide. This superoxide would then react with NO to form peroxynitrite. However, we saw no effect of superoxide dismutase on cytochrome *c* oxidation (results not shown). Such reactions, if they occur, cannot play a major role in the redox chemistry observed, at least at the lower levels of NO when SOD can compete effectively to prevent superoxide formation.

Some anomalies remain. If the only interaction of cytochrome *c* with the system is by reduction of NO_2_^•^, then the simple analysis of Fig. 7 predicts that increasing concentrations of ferrocytochrome *c* will not affect the overall rate of NO decay. However, at concentrations above 20 mM ferrocytochrome *c* has a significant ability to remove NO from aerobic solutions [12]; this is too large to be explainable by reversible binding of NO to the ferricytochrome *c* produced in the reaction. Related problems arise with the effects on NO decay rate of both non-redox ions, such as bicarbonate and phosphate, and inorganic reductants, such as ferrocyanide. It is possible that these molecules can access intermediates in the rate-limiting termolecular reaction of NO, NO, and O_2_, such as NO_3_^•^.

Sharpe and Cooper [12] also adduced some other evidence for the presence of nitroxyl anion production in the NO–ferrocytochrome *c* system, including nitrosylmyoglobin formation and the cross-linking of the reactive sulfhydryl in yeast cytochrome *c*. A mixture of ferric myoglobin with NO and ferrocytochrome *c* showed the characteristic nitrosylmyoglobin EPR signal, not seen in the presence of NO alone and otherwise requiring the addition of a strong reductant such as dithionite. Although detection of this signal is consistent with production of NO^−^, the reported yield was quite low. Other labile species may be capable of reducing metmyoglobin. Yeast ferrocytochrome *c* cross-linking (disulfide bridge formation) can, in our view, equally well be explained as an NO_2_^•^ effect (as with the oxidation of cysteine and glutathione). Finally, the Hepes radical can react with oxygen to produce superoxide [30], which in the presence of NO would be converted to peroxynitrite, possibly providing an alternative explanation for some of the other secondary oxidations observed. Thus, *contra* Sharpe and Cooper [12], we now agree with the thermodynamic findings that nitroxyl anion production requires a much stronger reductant-generating system than the ferro/ferricytochrome *c* redox couple [31]. Mitochondria cannot produce peroxynitrite via the reaction of nitroxyl anions with oxygen after the addition of NO to ferrocytochrome *c*.

When the distal methionine heme ligand is displaced, ferrocytochrome *c* can bind NO. There has been recent interest in this reaction due to the ability of cardiolipin to induce the displacement of this methionine and initiate proapoptotic oxidative chemistry [32]. NO can rapidly bind to cardiolipin cytochrome *c*[10] and attenuates this chemistry [9]. The work here supports the classical requirement for the removal of methionine ligation for NO to interact with the ferrous enzyme at physiological pH. Whether there is any physiological relevance of the ferrocytochrome *c* NO_2_^•^ scavenging that we report here remains to be seen, however. Cytochrome *c* resides in the mitochondrial inner membrane space, sandwiched between two membranes. NO autoxidation is likely to be high because of the concentrating effects of lipid membranes toward hydrophobic gases [28]. Nevertheless, even allowing for this, to probe mechanisms our study used unphysiologically high concentrations of NO. However, for the same reason, we used unphysiologically *low* concentrations of ferrocytochrome *c*. The cytochrome *c*/NO ratio is likely to be very high in the inner mitochondrial space. Under plausible physiological conditions (800 mM ferrocytochrome *c,* 0.5 mM NO) ferrocytochrome *c* is a very effective NO_2_^•^ trap. In fact all the NO_2_^•^ produced oxidizes cytochrome *c*, the system acting to qualitatively convert NO and ferrocytochrome *c* to nitrite and ferricytochrome *c* (Fig. 8d). Mitochondrial NO metabolism is also likely to be affected by other components of the respiratory chain, such as cytochrome *c* oxidase, which can very effectively convert NO to nitrite [33]. The relative importance of the reactions of cytochrome *c* with NO in such an aerobic system are therefore determined as much, if not more so, by competition with other molecules for NO and its oxidation products as by interactions with the steady-state concentrations predicted by the intrinsic NO autoxidation rates.
